# Evaluation of strain coverage of the multicomponent meningococcal serogroup B vaccine (4CMenB) administered in infants according to different immunisation schedules

**DOI:** 10.1080/21645515.2018.1537756

**Published:** 2019-01-02

**Authors:** Alessia Biolchi, Sara Tomei, Laura Santini, Jo Anne Welsch, Daniela Toneatto, Nikolaos Gaitatzis, Xilian Bai, Ray Borrow, Marzia Monica Giuliani, Elena Mori, Mariagrazia Pizza

**Affiliations:** a GSK, Siena, SI, Italy; b PATH, San Francisco, CA, USA; c GSK, Marburg, Germany; d Public Health England, Meningococcal Reference Unit, Manchester, UK

**Keywords:** meningococcal serogroup B, 4CMenB vaccine, strain coverage, serum bactericidal antibody assay, pooled sera, infant immunisation schedule

## Abstract

The 4-component vaccine 4CMenB, developed against invasive disease caused by meningococcal serogroup B, is approved for use in infants in several countries worldwide. 4CMenB is mostly used as 3 + 1 schedule, except for the UK, where a 2 + 1 schedule is used, and where the vaccine showed an effectiveness of 82.9%. Here we compared the coverage of two 4CMenB vaccination schedules (3 + 1 [2.5, 3.5, 5, 11 months] versus 2 + 1 [3.5, 5, 11 months of age]) against 40 serogroup B strains, representative of epidemiologically-relevant isolates circulating in England and Wales in 2007–2008, using sera from a previous phase 3b clinical trial. The strains were tested using hSBA on pooled sera of infants, collected at one month post-primary and booster vaccination. 4CMenB coverage was defined as the percentage of strains with positive killing (hSBA titres ≥ 4 after immunisation and negative baseline hSBA titres < 2). Coverage of 4CMenB was 40.0% (95% confidence interval [CI]: 24.9–56.7) and 87.5% (95%CI: 73.2–95.8) at one month post-primary and booster vaccination, respectively, regardless of immunisation schedule. Using a more conservative threshold (post-immunisation hSBA titres ≥ 8; baseline ≤ 2), at one month post-booster dose, strain coverages were 80% (3 + 1) and 70% (2 + 1). We used a linear regression model to assess correlation between post-immunisation hSBA data for each strain in the two groups; Pearson’s correlation coefficients were 0.93 and 0.99 at one month post-primary and booster vaccination. Overall, there is no evidence for a difference in strain coverage when 4CMenB is administered according to a 3 + 1 or 2 + 1 infant vaccination schedule.

## Introduction

Invasive meningococcal disease (IMD) is a rapidly progressing severe infectious disease caused by *Neisseria meningitidis*,^1^ which can lead to long-term sequelae in 11–19% of cases and has a case fatality ratio of around 10–15%.^2^

IMD incidence varies with age, presenting two peaks: one in infants and young children under five years of age, and another, lower one in adolescents and young adults.^3^ Six meningococcal serogroups (A, B, C, W, X and Y) account for most IMD cases, and their prevalence differs greatly by geographic regions.^4^ Serogroup B is frequently associated with outbreaks in high-income countries and was shown to be responsible for a high case fatality ratio (8.1%) in infants.^5^

The global use of meningococcal conjugate vaccines has drastically reduced disease incidence over the last two decades.^4^ The four-component meningococcal serogroup B recombinant vaccine (4CMenB, *Bexsero*, GSK) contains three recombinant antigens (factor H-binding protein, *Neisseria* adhesin A and Neisserial heparin-binding antigen) and the outer membrane vesicles of the New Zealand outbreak strain (NZ 98/254). The vaccine has already been shown to be immunogenic and well tolerated in all age groups,^6^ including infants, children and adolescents.^7,8^ Currently, 4CMenB is approved for use in infants in 39 countries including European countries, Canada, Australia, Chile, Uruguay, Argentina and Brazil^9^ as a 3 + 1 schedule, with primary doses administered in the first six months of life, and a booster dose at 12–23 months of age. In the United Kingdom (UK), 4CMenB was introduced in the national immunisation schedule in 2015 as a 2 + 1-dose series administered at 2, 4 and 12 months of age, which was considered cost-effective based on modelling data.^10^ A vaccine effectiveness of 82.9% was estimated against all serogroup B disease and 94.2% against vaccine-preventable group B strains, as measured during the first 10 months of the national immunisation programme, i.e. following the two-dose primary series.^11^

A previous clinical study comparing the administration of 4CMenB according to a 3 + 1 schedule (at 2.5, 3.5, 5, 11 months of age) with a reduced 2 + 1 (at 3.5, 5, 11 months of age) schedule in infants showed that the vaccine was safe and immunogenic.^7^ The immune response was evaluated by serum bactericidal antibody with human complement (hSBA) of individual sera against four indicator serogroup B strains; the bactericidal response induced by sera deriving from both immunisation schedules was comparable.^7^

The ability of 4CMenB to cover genetically diverse strains is predicted by the Meningococcal Antigen Typing System (MATS) assay, an enzyme-linked immunosorbent assay (ELISA) which measures the level of expression and the sequence diversity of each of the 4CMenB antigens in a given strain.^12^ Strains are predicted to be killed in the hSBA when their MATS relative potency (the ELISA reactivity compared to that of a reference serogroup B strain) is higher than the positive bactericidal threshold, specific for each antigen. This correlation has been defined by testing the killing ability of antibodies in infant sera derived from a 3 + 1 immunisation schedule on a panel of 57 genetically diverse strains.^12^

Due to the large number of circulating serogroup B strains, the MATS assay has been applied to strains isolated from a specific region or country to assess the potential breadth of coverage conferred by 4CMenB, and to anticipate the impact of vaccination on the local IMD burden. The estimated strain coverage was between 66% and 91%, depending on the epidemiology of serogroup B IMD in each country.^13^ However, prediction based on MATS can underestimate the strain coverage afforded by 4CMenB, because the method is based on single antigens and does not account for synergistic effects of antibodies directed against different antigens, nor for the contribution of antibodies against minor outer membrane antigens to complement-mediated bacterial killing. This has been clearly demonstrated by analysing a panel of 40 serogroup B strains representative of 535 strains isolated in England and Wales in 2007–2008, tested using the hSBA assay on pooled sera of infants (following a 3 + 1 vaccination series) and adolescents which showed higher strain coverage compared to MATS.^14^

This study compares the bactericidal activity of sera from a clinical trial carried out in infants receiving 4CMenB according to either the 3 + 1 or 2 + 1 vaccination schedule,^7^ to assess whether differences in the number of doses and the immunisation regimen would lead to differences in strain coverage. For this purpose, the panel of 40 previously characterised UK serogroup B strains was tested using the hSBA.

**Figure 1.**
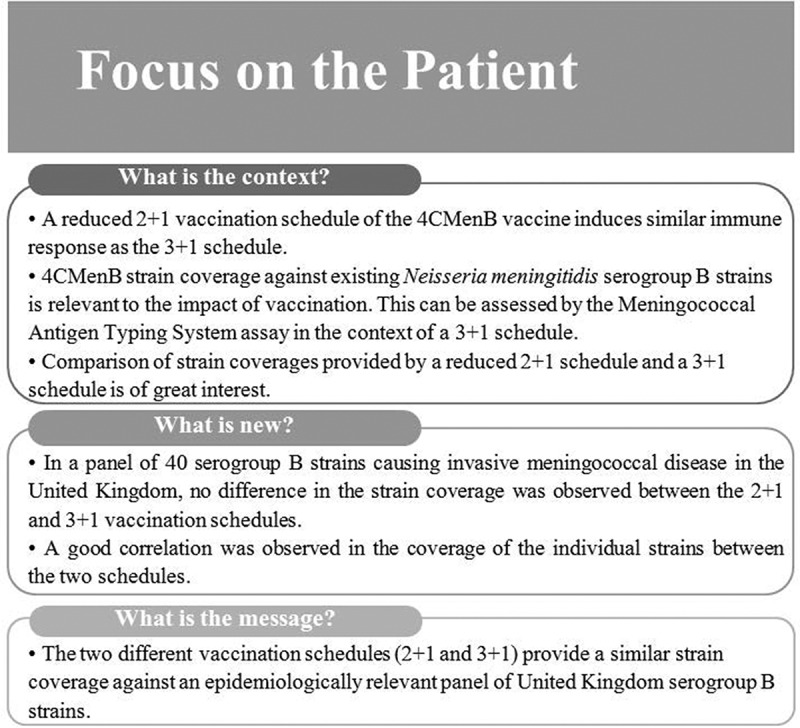
Focus on the patient section.

A summary contextualizing the results, the potential clinical research relevance and the impact of our study is described in the Focus on Patient Section (Figure 1).

## Results

**Figure 2.**
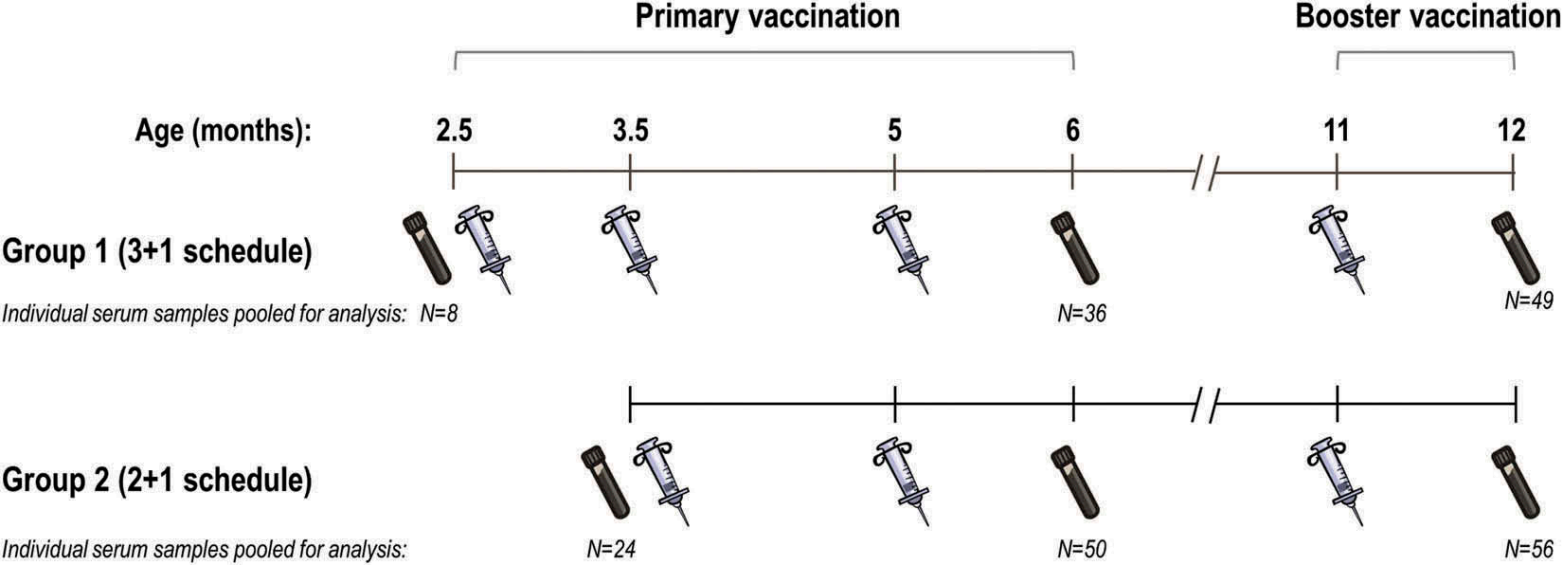
Study interventions and number of infants from whom sera was collected, by time point. N, number of infants with tested serum samples. Note: The test tube and syringe symbols indicate time points of blood draw and vaccination with 4CMenB, respectively.

The tested sera were collected from healthy infants under 5 months of age at enrolment, who received 4CMenB according to different schedules: either 3 + 1 (at 2.5, 3.5, 5 and 11 months of age; Group 1) or 2 + 1 (at 3.5, 5 and 11 months of age; Group 2).^7^ Pre-vaccination hSBA titres were assessed in pooled sera from 32 infants, regardless of the immunisation group. Serum samples were pooled from 36 infants in Group 1 and 50 infants in Group 2, at one month post-primary vaccination, and from 49 infants in Group 1 and 56 infants in Group 2, at one month post-booster vaccination (Figure 2).

At one month post-primary vaccination, strain coverage (defined as the percentage of serogroup B strains with positive killing, i.e. hSBA titres ≥ 4 and with a baseline < 2) was 40.0% (95% confidence interval [CI]: 24.9–56.7) for both groups. At one month post-booster vaccination, the percentage of strains killed in the bactericidal assay increased to 87.5% (95% CI: 73.2–95.8) for both vaccination schedules (Table 1).

**Table 1.** Percentage of strains covered by 4CMenB as evaluated by hSBA titres using different thresholds.

	Percentage of strains (95% confidence interval)	
	Group 1 (3 + 1 schedule)	Group 2 (2 + 1 schedule)
*hSBA titre ≥ 4 and baseline < 2*		
1 month post-primary vaccination	40.0 (24.9–56.7)	40.0 (24.9–56.7)
1 month post-booster vaccination	87.5 (73.2–95.8)	87.5 (73.2–95.8)
*hSBA titre ≥ 8 and baseline ≤ 2*		
1 month post-primary vaccination	25.0 (12.7–41.2)	25.0 (12.7–41.2)
1 month post-booster vaccination	80.0 (64.4–90.9)	70.0 (53.5–83.4)

hSBA, serum bactericidal antibody assay with human complement.

When assessing strain coverage using a more conservative threshold of an hSBA titre ≥ 8 and a baseline ≤ 2 positive killing was observed for 25% (95% CI: 12.7–41.2) of strains post-primary vaccination in both groups. At one month post-booster, positive killing was observed for 80% (95% CI: 64.4–90.9) and 70% (95% CI: 53.5–83.4) of strains in infants immunised with the 3 + 1 or 2 + 1 schedule, respectively (Table 1).

**Figure 3.**
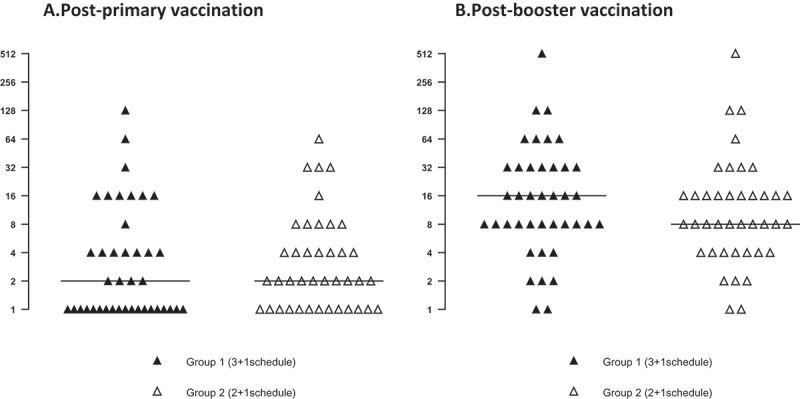
hSBA titres against individual serogroup B strains at one month post-primary (A) and booster (B) vaccination. hSBA, serum bactericidal antibody assay with human complement. Note: Horizontal lines represent the median value for hSBA titres in each group.

**Figure 4.**
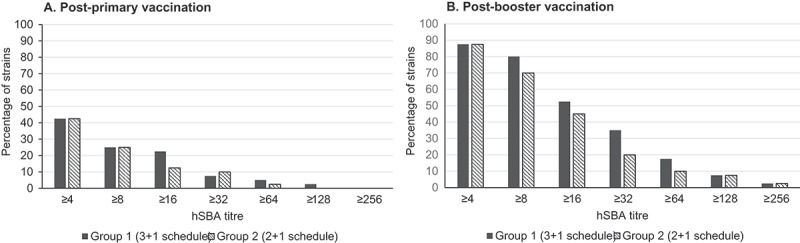
Distribution of hSBA titres against serogroup B strains at one month post-primary (A) and booster (B) vaccination. hSBA, serum bactericidal antibody assay with human complement.

Bactericidal titres of pooled sera from infants immunised with the two different schedules, tested against each of the 40 serogroup B strains, are presented in Figures 3 and 4. The bactericidal titres, as well as the number of strains killed at the same sera dilution, were comparable in the two groups, both at the post-primary and post-booster vaccination time points.

**Figure 5.**
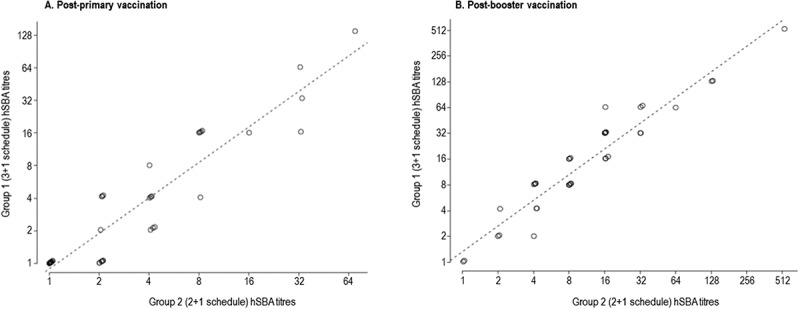
Correlation between hSBA titres of pooled sera in each group at one month post-primary (A) and booster vaccination (B). hSBA, serum bactericidal antibody assay with human complement.

A linear regression model was applied and fitted the data on hSBA titres of pooled sera for each individual strain in both groups at each time point (Figure 5). The calculated Pearson’s correlation coefficients were 0.93 and 0.99 at one month post-primary and booster vaccination, respectively.

## Discussion

This is the first study that compares the breadth of strain coverage conferred by two different immunisation schedules with 4CMenB, administered during infancy. Overall, there was no evidence for a difference in the percentage of strains covered by a 3 + 1 or 2 + 1 vaccination schedule in infants, using the hSBA to evaluate positive killing against a representative panel of 40 serogroup B invasive disease strains from England and Wales.

Pooled-sera hSBA titres have already been shown to predict individual seroprotection in infants and toddlers vaccinated with 4CMenB.^15^ A previous study demonstrated a strong correlation between pooled hSBA titres and the mean of the individual titres for sera composing the pool, and showed that hSBA performed on pooled sera predicts individual seroprotection.^15^ The serological correlate of protection against meningococcal disease, an hSBA titre of ≥ 4 was used to assess the immune response to vaccination for the licensure of 4CMenB in Europe.^16,17^ In our study, positive killing of serogroup B strains was defined as reaching the hSBA titre of ≥ 4 and a ≥ 4-fold increase in titres compared to baseline values, simultaneously. hSBA killing was observed for a high percentage of strains (87.5%) after the completion of either the 3 + 1 or 2 + 1 vaccination series with 4CMenB. When a more conservative threshold was applied (hSBA titre ≥ 8 and baseline ≤ 2), strain coverage at one month post-booster vaccination was slightly different between the two groups, with estimates of 80% and 70% assessed for the 3 + 1 and 2 + 1 immunisation schedules, respectively. These data are consistent with results previously obtained for pooled sera derived from infants vaccinated according to 3 + 1 schedules and from adolescents receiving two doses of 4CMenB, using the same panel of tested strains, for which a strain coverage of 88% was estimated.^14^

Of note, the difference in strain coverage observed between the two groups was approximately 10% when using a more conservative threshold. Due to the descriptive nature of the study design, statistical power was not sufficient to assess differences in coverage based on 40 strains. We cannot therefore exclude the possibility that one additional dose administered in infants could have an impact on antibody affinity, resulting in the more efficient killing of certain strains.

Strain coverage by 4CMenB was comparable between the two immunisation schedules, and was lower at the post-primary (40%) than at the post-booster (87.5%) time point in both groups. When compared to the 2-dose vaccine effectiveness of 82.9% measured during the first 10 months of the mass vaccination program with 4CMenB in the UK,^11^ the 40% strain coverage related to post-primary immunisation suggests that, although hSBA is an excellent predictor of protection, it largely underestimates the real protection from disease. This comparison may be hindered by the fact that our analysis was conducted using strains circulating in 2007–2008, while the effectiveness data is reported for 2015. Changes in the genetic diversity of serogroup B strains -and also for the MATS-predicted coverage afforded by 4CMenB- have already been reported in the UK.^18^ An hSBA titre ≥ 4 as a correlate of protection was postulated in the 1960’s and is now known to underestimate immunity,^19^ because while a positive result in hSBA indicates protection against meningococcal disease, a negative hSBA result is not necessarily an indication of susceptibility.^17^ As MATS underestimates positive results in hSBA, it is therefore an even more conservative predictor of protection than hSBA.^14^ Nevertheless, the differences between the percentage of strains killed in hSBA after primary and booster vaccination show that the booster dose improves 4CMenB strain coverage, further suggesting that compliance with the full vaccination schedule is important in achieving a broader protection against serogroup B strains.

A linear regression model fitted well the hSBA data from each group against individual serogroup B strains, confirming that the 3 + 1 and 2 + 1 immunisation schedules provide comparable coverage against serogroup B strains.

For both schedules, hSBA titres < 4 were observed for five out of the 40 serogroup B strains following the booster dose, in line with pooled-sera hSBA data previously obtained for infants using the same strain panel.^14^ The clinical relevance of this observation is not clear, as an hSBA titre ≥ 4 is defined as the correlate of protection although titres below this threshold do not necessarily indicate susceptibility to disease caused by the strain in question.^17^

The study’s main strength was the use of a panel of 40 serogroup B disease isolates, selected as representative of the 525 serogroup B disease strains isolated in Wales and England in 2007−2008. The results of this study may not match the coverage of serogroup B strains circulating between 2011 and 2014, when the infant sera were collected, given the known changes over time in the genetic diversity of meningococcal isolates, and in particular of serogroup B strains.^18,20^ Future studies evaluating the coverage in a panel of strains isolated in more recent years, after the implementation of 4CMenB vaccination, will be of great interest. Nevertheless, this does not impact the comparison between the two schedules. Second, the 2 schedules used in our analyses differ slightly in the timing of vaccine doses from the schedules currently approved for use, and this might constitute a further limitation of our study. Of note, the 2.5, 3.5, 5, 11 month-schedule was already shown to be well tolerated and to induce similar immune responses to the licensed 3 + 1 schedule, while data obtained for the 3.5, 5, 11 month-vaccination series supported the recommendation for the introduction of the reduced 2 + 1 schedule in the UK national immunisation programme.^7^ Therefore, we believe that the different timing of doses does not impact the generalisation of our comparison to the licensed schedules. In addition, the number of strains analysed was too low to assess statistical differences, and all analyses were descriptive only. However, this study provides an interesting outlook into the strain coverage afforded by the two different immunisation schedules. Future studies will help to evaluate whether differences in the number of doses have a significant impact on antibody persistence and their strain cross-coverage potential.

## Conclusions

Strain coverage of 4CMenB assessed using a threshold of hSBA titre ≥ 4 against an epidemiologically relevant and representative panel of 40 invasive UK serogroup B strains was 87.5% at one month after the booster dose, regardless of the primary series received (two or three doses). Our results showed no evidence for a difference in the breadth of coverage provided by the 4CMenB vaccine when administered according to a 3 + 1 schedule or a reduced 2 + 1 schedule, as currently used in the UK national infant immunisation programme.

## Methods

### Study design

This study was carried out using data from a large phase 3b, multicentre, clinical trial (NCT01339923) conducted between 2011 and 2014, in which healthy < 5-month-old infants were enrolled to receive 4CMenB according to two different schedules: at 2.5, 3.5, 5 and 11 months of age (3 + 1; Group 1) or at 3.5, 5 and 11 months of age (2 + 1; Group 2),^7^ (Figure 2). In the current study, a re-analysis of hSBA assessments was conducted using pooled sera collected from infants only from the nine centres in Spain, from whom an appropriate volume of blood was available and informed consent to re-use blood samples was obtained by parents/legally acceptable representatives. Full criteria for the inclusion/exclusion of study participants in the phase 3b trial were previously described in detail.^7^

The study was conducted in accordance with the principles of Good Clinical Practice and the Declaration of Helsinki.

### hSBA assay

The hSBA assay was performed at the GSK Research Centre in Siena, Italy, using pooled sera from children in each group at one month post-primary and post-booster vaccination. At baseline, sera from both groups were pooled and tested, while at one month post-primary and post-booster vaccination, sera from each group were pooled and tested separately. Vaccine coverage against a representative panel of serogroup B strains was defined as the percentage of strains for which the hSBA was positive, i.e. for which an hSBA titre ≥ 4 and a baseline < 2. The same endpoint was evaluated in a more conservative manner, using an hSBA titre ≥ 8 and baseline ≤ 2. The assay used has been previously described.^14^

### Selected strains

The panel of invasive disease isolates represented a subset of 40 serogroup B strains, previously selected based on their MATS phenotype and their genotypic profile from a total of 535 IMD isolates from England and Wales, collected between July 2007 and June 2008.^14^ The selection was performed using a stratified proportional sampling method and no significant bias was detected between the 40-strain subset and the total strain set when comparing the frequency distribution of MATS antigen phenotypes (p = 0.999), the distribution of MLST genotypes (p = 0.972), fHbp genotypes (p = 0.576), and NHBA genotypes (p = 0.619). The selection procedure and characteristics of the 40 serogroup B isolates have been previously described in detail^14^ and have been reported in Figure S1.

### Study objectives

The primary objective of this study was to compare the coverage of 4CMenB against the panel of representative serogroup B strains in terms of bactericidal activity of pooled sera from infants immunised with the 3 + 1 versus the 2 + 1 schedule, at one month post-booster vaccination.

Secondary objectives included the evaluation of 4CMenB coverage at each post-vaccination time point and coverage comparison as assessed in Group 2 (receiving the 2 + 1 schedule), with that of Group 1 (receiving the 3 + 1 schedule) at six months of age.

### Statistical analyses

The number and percentage of serogroup B strains covered by 4CMenB were summarised for each group and at each post-vaccination visit, with 95% CIs computed using the Clopper-Pearson method.^21^

To show the relationship between the two immunisation schedules, a linear regression model was fitted to log_2_-transformed hSBA titres against each serogroup B strain in the two groups, using the least-squares method. The Pearson correlation coefficient was also calculated, post-primary and post-booster vaccination.

All analyses were performed using STATA.

## Abbreviations

4CMenB: four-component meningococcal serogroup B recombinant vaccine
CI: confidence interval
ELISA: enzyme-linked immunosorbent assay
hSBA: SBA assay with human complement
IMD: invasive meningococcal disease
MATS: Meningococcal Antigen Typing System
SBA: serum bactericidal activity
UK: United Kingdom

## Trademark

*Bexsero* is a trademark owned by the GSK group of companies.
