# Supplementary material for: Evaluation of strain coverage of the multicomponent meningococcal serogroup B vaccine (4CMenB) administered in infants according to different immunisation schedules
Source: Hum Vaccin Immunother. 2019 Jan 2;15(3):725–31. doi: 10.1080/21645515.2018.1537756 (PMC6605712; doi:10.1080/21645515.2018.1537756)
Supplement: Supplemental Material [file khvi-15-03-1537756-s001.docx]

**Supplementary Material**

# Evaluation of strain coverage of the multicomponent meningococcal serogroup B vaccine (4CMenB) administered in infants according to different immunisation schedules

Alessia Biolchi,^a^ Sara Tomei,^a^ Laura Santini,^a^ Jo Anne Welsch,^b^ Daniela Toneatto,^a^ Nikolaos Gaitatzis,^c^ Xilian Bai,^d^ Ray Borrow,^d^ Marzia Monica Giuliani,^a^ Elena Mori,^a^ Mariagrazia Pizza^a^

# Affiliation:

^a^ GSK, Via Fiorentina, 1, 53100 Siena SI, Italy; [alessia.x.biolchi@gsk.com](mailto:alessia.x.biolchi@gsk.com), [sara.x.tomei@gsk.com](mailto:sara.x.tomei@gsk.com); [laura.x.santini@gsk.com](mailto:laura.x.santini@gsk.com); [daniela.x.toneatto@gsk.com](mailto:daniela.x.toneatto@gsk.com); [marzia.m.giuliani@gsk.com](mailto:marzia.m.giuliani@gsk.com); [elena.x.mori@gsk.com](mailto:elena.x.mori@gsk.com); [mariagrazia.x.pizza@gsk.com](mailto:mariagrazia.x.pizza@gsk.com)

^b^ PATH, 600 California Street, 11th floor, San Francisco, CA 94108, United States; [jwelsch@path.org](mailto:jwelsch@path.org)

^c^ GSK, Emil-von-Behring-Strasse 76, 35041 Marburg, Germany; [nikolaos.x.gaitatzis@gsk.com](mailto:nikolaos.x.gaitatzis@gsk.com)

^d^ Public Health England, Meningococcal Reference Unit, Clinical Sciences Building 2, Manchester Royal Infirmary, Oxford Road, Manchester, M13 9WL, United Kingdom; [xilian.bai@phe.gov.uk](mailto:xilian.bai@phe.gov.uk); [ray.borrow@phe.gov.uk](mailto:ray.borrow@phe.gov.uk)

# Contact information

Mariagrazia Pizza, GSK, Via Fiorentina, 1, 53100 Siena SI, Italy; [mariagrazia.x.pizza@gsk.com](mailto:mariagrazia.x.pizza@gsk.com)

**Figure S1.** Distributions of MATS antigen phenotypes (A), MLST genotypes (B), NHBA genotypes (C) and fHbp genotypes (D) in the total and subset of serogroup B isolates identified in England and Wales


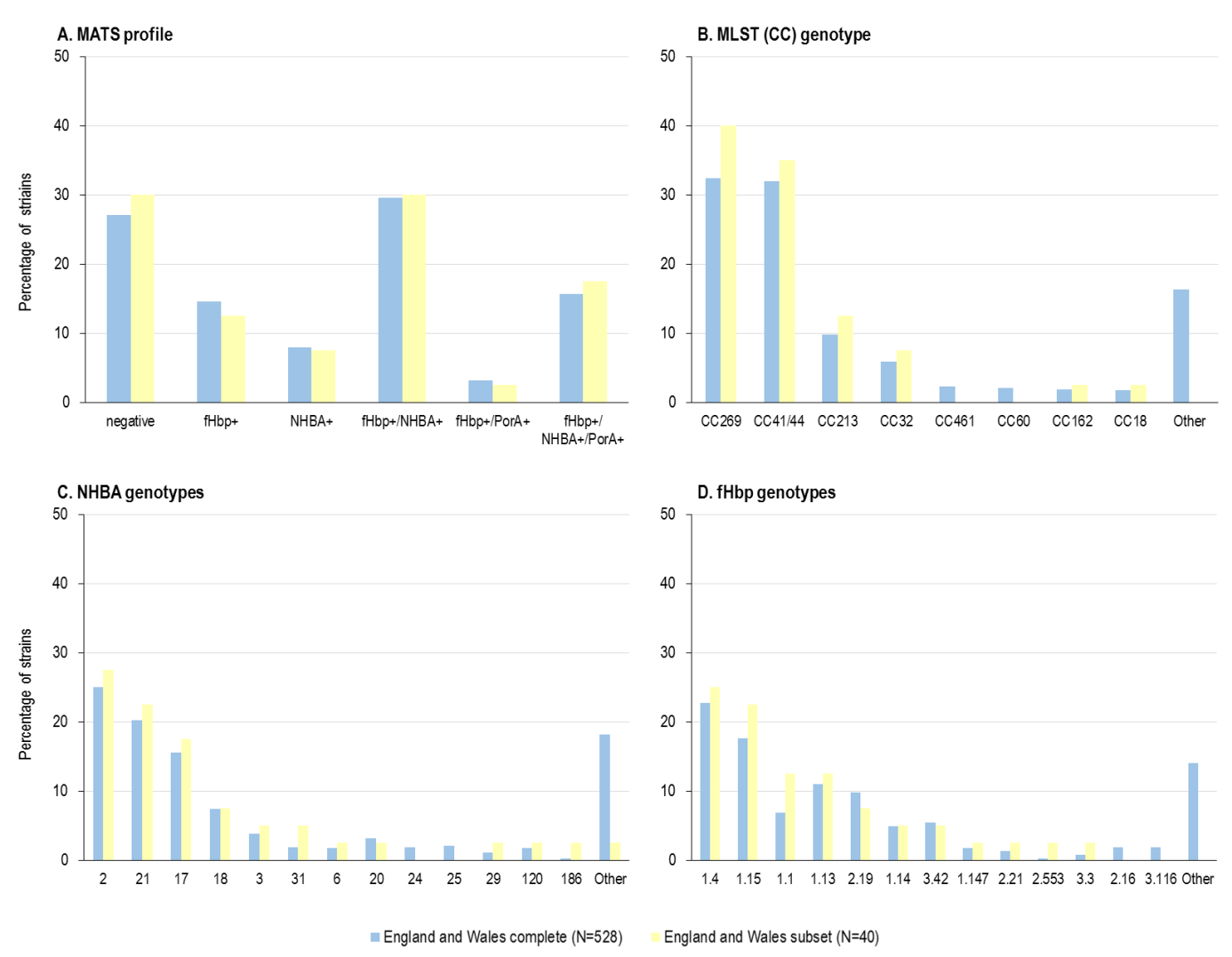


MATS, meningococcal antigen typing system; MLST, multilocus sequence typing; CC, clonal complex; NHBA, Neisserial heparin binding antigen; fHbp, factor H binding protein; N, number of strains.
